# Longitudinal Trajectories of Housing Quality and Their Associations with Elderly Depression: A Prospective Cohort Study Based on the China Longitudinal Aging Social Survey, 2018–2023

**DOI:** 10.3390/bs16101920

**Published:** 2026-10-16

**Authors:** Chen Jiang, Junfeng Jiang, Yugao Tang

**Affiliations:** 1 School of Ethnology and Sociology, Minzu University of China, Beijing 100081, China; 21400064@muc.edu.cn; 2 School of Sociology, Central China Normal University, Wuhan 430079, China; tangyugao@mails.ccnu.edu.cn

**Keywords:** housing quality, depression, group-based trajectories, Chinese older adults

## Abstract

Prior studies have demonstrated the crucial role of housing quality in the mental health of older adults. However, there remains insufficient evidence regarding how longitudinal trajectories of housing quality relate to elderly depression. Based on the China Longitudinal Aging Social Survey conducted from 2018 to 2023, this study used the group-based trajectory model to identify distinct longitudinal trajectories of housing quality (measured using nine housing characteristics) in Chinese older adults and subsequently examined their associations with depressive symptoms, as well as potential mediations and urban–rural differences. Three group-based trajectories, including “consistently high-level”, “moderate-fluctuating growth”, and “low-fluctuating growth”, were yielded. Compared with the “low-fluctuating growth” trajectory, older adults in the “consistently high-level” trajectory exhibited significantly lower depression levels (β = −0.484, *p* < 0.001), whereas those in the “moderate-fluctuating growth” trajectory exhibited no significant changes. This association did not differ significantly between urban and rural areas. Mechanism analysis indicated that, in rural areas, better physical health, stronger social support networks, and higher levels of social adaptation served as important mediators that linked elevated housing quality with reduced depression. By contrast, in urban areas, social support networks and social adaptation did not demonstrate significant mediating effects, and better physical health may mask the association between housing quality and elderly depression. These findings suggest that we should avoid staged declines in housing quality among the elderly and pay attention to urban–rural differences in the mechanisms linking housing quality trajectories with elderly depression.

## 1. Introduction

Depression, one of the most common mental disorders in elderly populations (McElroy & Walsh, 2023), not only severely impairs these individuals’ quality of life but also demonstrates significant associations with physical illnesses, cognitive decline, and suicide risks (Charlson et al., 2016; Kang et al., 2019; Xu & Cao, 2025). Due to a rapid social transition in recent decades, China’s elderly population is facing particularly severe challenges regarding mental health (Jiang & Deng, 2026; Li & Zhang, 2014). Extensive research has established robust correlations between housing quality and elderly depression, indicating that poor housing conditions are positively related to elderly depressive risks (J. H. Lee, 2024; Pevalin et al., 2017), while housing quality improvement is associated with elevated social capital and life satisfaction, thereby mitigating depressive symptoms (Cheng et al., 2025; Sanders et al., 2025a; Song et al., 2025).

Existing studies have primarily adopted a static perspective to investigate the association between housing quality levels and elderly depressive symptoms. However, from a dynamic perspective, older adults with similar housing quality levels may exhibit completely distinct temporal trajectories in housing quality. Obviously, a single cross-sectional exposure cannot distinguish between individuals who have consistently experienced good housing conditions and those whose housing conditions have recently improved or deteriorated. There remains insufficient evidence clarifying how different trajectories of housing quality uniquely relate to depressive symptoms in older adults. Additionally, Chinese residents have recently experienced dramatic changes in housing conditions, and the processes of housing quality change between urban and rural areas are distinct (Liang, 2020; Xiao & Selvaratnam, 2024). The association between housing quality trajectories and elderly depression may therefore present specific urban–rural disparities. Accordingly, this study employs a nationally representative dataset to investigate the potential trajectories of housing quality in older adults and their associations with elderly depression, as well as their urban–rural disparities in China, aiming to provide empirical foundations for targeted housing and public health policies.

## 2. Literature Review and Research Hypotheses

### 2.1. Housing Quality and Elderly Depression

Regarding the relationship between housing quality and mental health, scholars have proposed multiple theoretical explanations. The learned helplessness theory posits that prolonged exposure to uncontrollable adverse environments without escape opportunities will gradually induce helplessness, leading to social withdrawal behaviors and subsequent depression. As an expensive and immutable asset, terrible housing conditions can readily become settings that trigger learned helplessness (J. H. Lee, 2024). The relative deprivation theory suggests that inadequate housing security constitutes psychological deprivation, which may provoke depression, anxiety and feelings of insecurity (Szabo et al., 2018). By contrast, an ecological stress process model conceptualizes poor housing quality as a meso-level life stressor that elevates mental health risks (Liang et al., 2017). Collectively, these theoretical perspectives converge to a common conclusion that substandard housing conditions are related to deteriorated mental health.

Substantial empirical evidence also demonstrates that housing quality serves as a critical determinant of mental health, particularly depressive symptoms, with this influence consistently observed across diverse populations. For instance, some studies document significant associations between poor housing conditions and elevated depression risks among low-income populations and displaced people (Garg et al., 2022; Schilz et al., 2023). Both poor housing quality and housing instability show significant correlations with elevated depressive symptoms in adolescents (Baek & Yoon, 2025; Morganti et al., 2022; Zhou et al., 2022). Both cross-sectional and longitudinal associations are observed between substandard housing conditions and elevated depressive symptoms in females (Sanders et al., 2025b). Among general adult populations, housing quality is also significantly associated with most mental health indicators, including depression, anxiety, and cognitive function (Pineda et al., 2021; H. Yuan, 2025).

Housing quality demonstrates particularly strong associations with elderly depression. A longitudinal study investigating New Zealand seniors finds that homeowners exhibit lower levels of depression than renters (Szabo et al., 2018). Research on Colombian elderly individuals has identified significant associations between inferior housing quality and elevated depression scores (Osuna et al., 2026). Also, studies focusing on Chinese older adults confirm housing quality as an important factor relating to depression in both urban and rural areas (Song et al., 2025). Furthermore, among rural seniors, housing conditions serve as crucial predictors of depression trajectories, with sustained occupancy of good housing conditions being linked to continuously lower depression levels (Liang, 2020). However, regrettably, no research has yet investigated how dynamic changes in housing quality are associated with depression among older adults. Accordingly, the following research hypothesis was proposed:

**H1.**  *Trajectories of high-level housing quality are significantly associated with reduced depression among older adults.*

### 2.2. Urban–Rural Disparities

The association between housing conditions and mental health exhibits significant variation across different social contexts (McElroy & Walsh, 2023). In China, urban–rural disparities in housing quality and its longitudinal changes have been extensively documented, which may lead to substantial heterogeneity in the association between trajectories of housing quality and elderly depression across urban and rural areas.

On the one hand, Chinese urban and rural residents have experienced persistent gaps in housing conditions over extended periods, with distinct driving mechanisms for these changes. Over the past decade or so, improvements in urban housing quality have been primarily driven by old town and shantytown renovation and housing market reforms, while improvements in rural housing quality have mainly resulted from governmental emphasis on housing conditions during poverty alleviation (2015–2020) and rural revitalization strategies (since 2017), particularly through dilapidated house reconstruction projects (Liang, 2020; Zhang et al., 2023). Urban residential improvements are predominantly market-driven, while rural counterparts are policy-driven (Liang, 2020; Xiao & Selvaratnam, 2024). These divergent driving processes might generate differential associations between housing quality trajectories and elderly depression across urban–rural contexts. Furthermore, given the historical pattern where rural residents consistently maintain lower levels of housing quality than urban dwellers, improvements in housing quality could potentially yield greater marginal contributions to psychological health among rural older adults.

On the other hand, social withdrawal theory posits that substandard housing conditions can reduce social interactions and adaptive capacities, triggering social withdrawal phenomena that subsequently exacerbate psychological distress (Song et al., 2025; Wells & Harris, 2007). Liang (2020) further emphasized that rural China features closer interpersonal relationships where housing serves as a crucial venue for social interactions while simultaneously carrying psychosocial attributes of “flaunt”, thereby strengthening the association between elevated housing quality and declined depression.

Although some evidence from other countries suggests poor housing conditions are related to elevated depression risks among urban but not rural older adults (Osuna et al., 2026), more empirical studies from China reveal significant associations between substandard housing conditions and increased depression risks among rural elderly individuals (Liang, 2020), with this association being notably stronger in rural older adults compared to their urban counterparts (Song et al., 2025). Accordingly, the following hypothesis was proposed:

**H2.**  *The association between trajectories of high-level housing quality and reduced depression levels is stronger in rural older adults compared to their urban counterparts.*

### 2.3. Physical Health, Social Support Networks, and Social Adaptation as Mediators

The ecological stress process model posits that environmental factors, as external stressors, exert critical influences on individual mental health by affecting the resources and capabilities required for social actions (Snedker & Herting, 2016). For older adults, action-related resources primarily encompass their own physical health status and external social support networks, while capabilities mainly refer to adaptive capacity to external environments. Thus, as a meso-level environmental factor, housing quality promotion may relate to depressive symptoms in older adults through physical health, social support networks, and social adaptation abilities.

Regarding physical health, poor housing conditions—such as dim lighting, dampness, poor ventilation, lack of home equipment, and indoor air pollution—usually relate to risks of injury and chronic disease and declined physical health among older adults (Chen et al., 2024; Zhang et al., 2023). Physical functional decline and deteriorating physical health status are confirmed to be significant risk factors for elderly depression, as physical impairments can restrict older adults’ daily activities and social engagement, thereby triggering mental health problems (Wang et al., 2024; M. Y. C. Wong et al., 2023).

A house usually serves as a physical space facilitating social interactions. High-quality housing conditions contribute to neighborhood interactions, which construct and maintain broader social support networks. Conversely, poor housing conditions may induce social withdrawal behaviors, reduce social participation, and consequently exacerbate psychological distress (Liang, 2020; Wells & Harris, 2007). Empirical evidence demonstrates that housing quality is related to social capital, with more social capital contributing to reduced depression levels (Song et al., 2025). Additionally, neighborhood quality has been found to buffer the negative association between poor housing conditions and good mental health (C. S. M. Wong et al., 2024). Recent Canadian evidence further revealed that higher levels of housing satisfaction can mitigate psychological distress by increasing residents’ social support (Taylor et al., 2026).

Furthermore, age-friendly housing renovation is a critical component of eldercare policies. Renovations—including installing handrails, improving lighting systems, and creating barrier-free access—can significantly enhance these individuals’ living capabilities and sense of security, enabling better adaptation to age-related functional limitations and the change in the world (M. Yuan, 2022). Empirical research indicates that good social adaptation abilities can strengthen self-efficacy and life satisfaction while reducing feelings of frustration and helplessness, thereby reducing depression risks (Jiang & Luo, 2024). Conversely, poor housing quality can lead to a diminished capacity to adapt to stress in elderly populations, generating persistent mental health challenges (Roberts et al., 2025). Accordingly, the following research hypotheses were proposed:

**H3a.**  *Physical health mediates the association between trajectories of high-level housing quality and reduced elderly depression.*

**H3b.**  *Social support networks mediate the association between trajectories of high-level housing quality and reduced elderly depression.*

**H3c.**  *Social adaptation mediates the association between trajectories of high-level housing quality and reduced elderly depression.*

## 3. Data and Methods

### 3.1. Data Source

The data used in this study were derived from the China Longitudinal Aging Social Survey (CLASS) conducted in 2018, 2020, and 2023. CLASS is a nationwide large-scale social survey that systematically collects socioeconomic data on Chinese older adults, providing critical evidence for addressing aging-related issues in China. The baseline survey was implemented in 2014, followed by four waves of follow-up investigations conducted in 2016, 2018, 2020, and 2023. Using a stratified multistage probability sampling method, the CLASS collects data across 28 provincial administrative regions through household interviews. A map-address-based sampling frame is used to sample household units, with subsequent within-household sampling targeting individuals aged 60 or older. One respondent per selected household is interviewed, while quality control strategies such as field supervision, remote data analysis, and telephone verification are adopted to ensure data reliability. Since housing quality information was not collected in 2014 and 2016, this study utilized three waves of data (2018, 2020, and 2023) for analysis.

**Figure A1:**
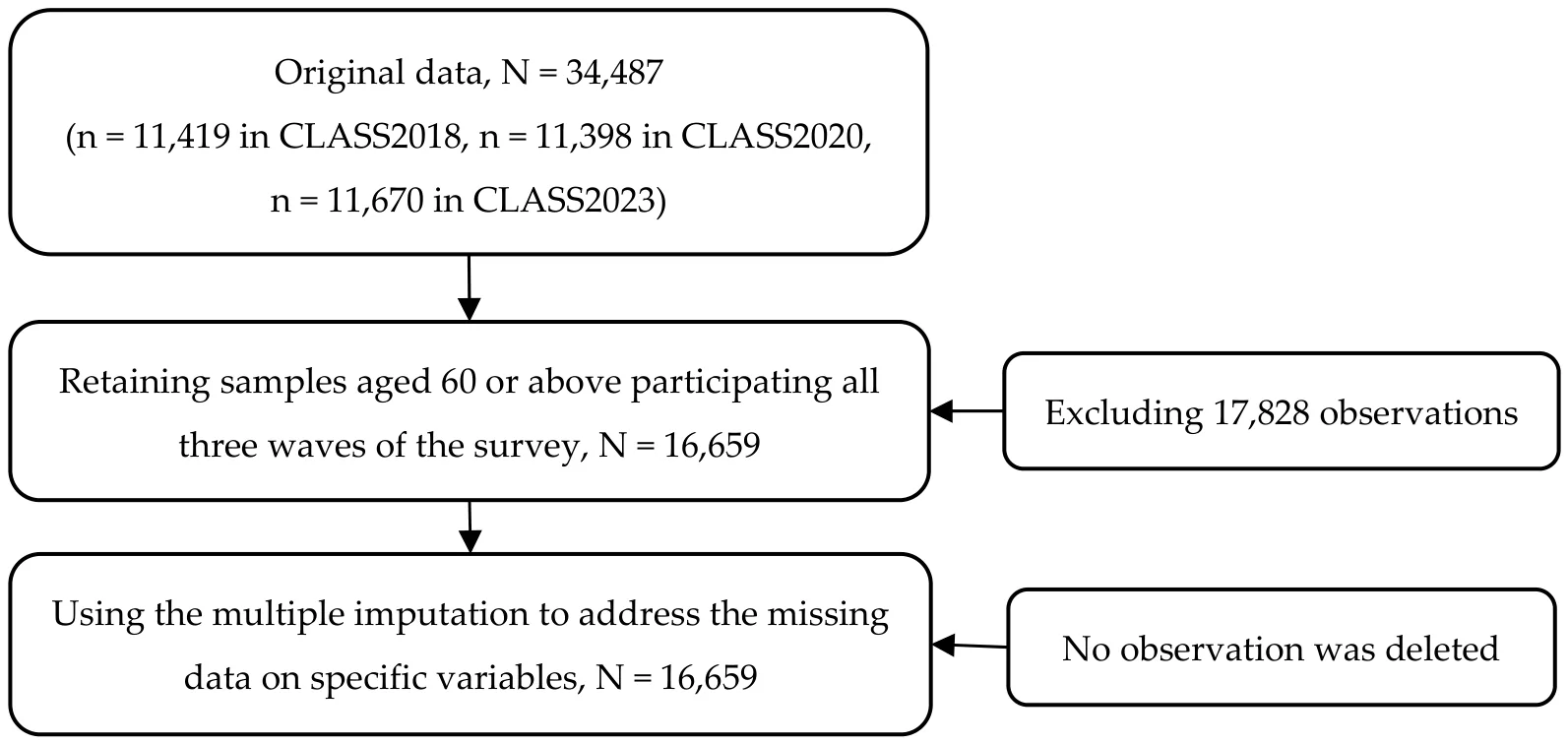
Detailed process of data cleaning.

In the original dataset, CLASS2018 collected 11,419 samples, CLASS2020 collected 11,398 samples, and CLASS2023 collected 11,670 samples. The inclusion criteria for sample selection primarily consisted of two requirements: first, age range of 60 years or older; second, participation in all three waves of the survey, as this was required for estimation of housing quality trajectories over time. Finally, a multiple imputation strategy was applied to handle missing data in specific variables^1^. Based on these criteria, a total of 5553 valid samples (comprising 16,659 person-times) were retained. Details regarding the data cleaning process are presented in Figure A1.

### 3.2. Variables

Explanatory variable: trajectories of housing quality. The CLASS collected data on whether respondents’ dwellings had the following facilities: bathroom, indoor toilet, emergency call device, handrails, running water, natural gas, the Internet, and the presence of dim lighting and uneven floors. These measures were highly similar to those of Liang (2020). These nine items (Cronbach’s α = 0.655)^2^ were summed to create a housing quality index ranging from 0 to 9. Subsequently, this study estimated trajectories of housing quality using the group-based trajectory model (GBTM) and used them as the explanatory variable. Detailed estimation results regarding the trajectories of housing quality are presented in Section 4.

Outcome variable: elderly depression in CLASS2023. Following Jiang and Deng’s study (2026), the short-form Center for Epidemiologic Studies Depression Scale (CES-D-9) was used to measure elderly depression. This scale contained nine specific items asking respondents whether they experienced the following feelings in the past week: being in a good mood, lonely, sad, having a good time, unwilling to eat, sleeping terribly, feeling useless, having nothing to do, and having a lot of fun in life. Each item was measured on a three-point scale ranging from “none” to “often” (Cronbach’s α > 0.70). A composite depression score was calculated by summing the nine items for a total ranging from 0 to 18, after reverse-scoring items 1, 4, and 9; higher scores reflected higher levels of depression.

Mediation variables included physical health, social support networks, and social adaptation in CLASS2020. Physical health was measured by three indicators: self-rated physical health, presence of physical pain, and chronic disease condition. Specifically, self-rated physical health assessed respondents’ perceived physical health level over the past week using a five-point Likert scale ranging from “0 = very unhealthy” to “4 = very healthy”. The variables for physical pain (presence = 0; absence = 1) and chronic disease condition (presence = 0; absence = 1) were binary variables. We calculated a composite physical health variable by summing the standardized self-rated physical health score (divided by 4) with the other two binary indicators, resulting in a continuous variable ranging from 0 to 3, where higher values indicated better physical health status. The social support networks variable consisted of six specific items (He & Yan, 2022; Jiang & Deng, 2026), including the ability to meet or contact family/relatives (friends) per month, how many family/relatives (friends) with which one could safely talk about personal matters, and how many family/relatives (friends) could provide help when needed. The options included “0 = none”, “1 = 1”, “2 = 2”, “3–4 = 3”, “5–8 = 4”, and “9 or more = 5” (reliability coefficient α > 0.70). The above six items were added to obtain a social support networks variable ranging from 0 to 30, with higher scores representing greater social support. Social adaptation comprised eight specific items (Wen & Lian, 2025) assessing respondents’ agreement with statements about their current situation: willingness to participate in community work; desire to contribute more to society; enjoyment of learning; perception of being a socially useful person; difficulty adapting to rapid social changes; increasing difficulty accepting diverse viewpoints; challenges coping with new policies; and perceived disadvantages of changes in modern society for the elderly. Each item was measured on a five-point Likert scale ranging from “completely disagree” to “completely agree” (Cronbach’s α > 0.70). After reverse-coding the last four items, these eight items were summed to yield a social adaptation variable ranging from 0 to 32, where higher scores represented better social adaptation.

Control variables included sex (male or female), age (≥60), residence (urban or rural), schooling year, marital status (married or unmarried—including never married, divorced, and widowed), and living alone (yes or no) in the baseline survey. These variables have been extensively validated to be closely associated with the risk of depression in older adults (Song et al., 2025; Liang, 2020). Residence also served as a key grouping variable. Detailed information can be seen in Table 1.

### 3.3. Research Methods

The present study first used the *t*-test and chi2 test to examine the urban–rural differences in all other variables used, and the related effect sizes were also reported based on Cohen’s d or Cohen’s w. Then, the GBTM was used to identify distinct trajectories of housing quality over time in Chinese older adults. Based on a three-wave longitudinal survey design, a quadratic functional form was specified to optimally capture trajectory patterns. Subsequently, the ordinary least squares (OLS) regression model was applied to examine whether significant differences exist in depression levels among older adults belonging to distinct trajectory groups. The Karlson–Holm–Breen (KHB) method for mediation decomposition (Karlson et al., 2012) was applied to examine the mediating roles of physical health, social support networks, and social adaptation in the association between housing quality trajectory and elderly depression. The KHB method has been widely used in prior studies and can be used in both linear and nonlinear models (Jiang & Luo, 2024; Karlson et al., 2012).

In robustness analyses, we first used the propensity score matching (PSM) approach to control for potential selection bias, thereby further verifying the observed depression differences across trajectory groups. Second, depression outcome was treated as a binary variable with the binary Logit model estimating associations between housing quality trajectories and geriatric depressive symptoms. Third, the fixed-effects (FE) model was employed to account for time-invariant individual heterogeneity, supplementing evidence regarding the interrelationship between changes in housing quality and variations in elderly depression. Finally, the causal mediation analysis was used to evaluate the robustness of prior KHB mediation effect estimates. All data cleaning and statistical analyses were performed using Stata version 18.0^3^, with statistical significance determined at α = 0.05. All statistical inference analyses were weighted to ensure the generalizability of the estimates.

## 4. Results

### 4.1. Urban–Rural Disparities in Basic Characteristics of Elderly Samples

Table 1 shows that there were significant urban–rural differences in the elderly sample across demographic characteristics, health status, social support networks, social adaptation, and housing quality. Urban seniors exhibited significantly more educational attainments, a higher proportion of being married, and better physical health and social adaptation compared to their rural counterparts. Conversely, rural seniors showed significantly higher proportions of living alone, worse physical health, and higher levels of social support networks. The housing quality of urban seniors was substantially superior to that of rural ones, with the former also demonstrating significantly lower depression levels than the latter. Particularly, it was observed that only the urban–rural disparities in schooling year and housing quality exhibited medium to large effect sizes (Cohen’s d = 0.614 and 0.969, respectively), while other urban–rural disparities only showed small effect sizes.

### 4.2. Estimation of Housing Quality Trajectories

Based on the GBTM, Table 2 indicates that as the number of fitted trajectories increased, the goodness-of-fit measured by AIC and BIC demonstrated continuous improvement, and the *p*-values of the Lo–Mendell–Rubin likelihood ratio test (LMR-LRT) consistently remained below 0.001, which appears to indicate that model fit continuously improves with increasing numbers of trajectory groups (Nagin, 2005; Nagin & Odgers, 2010). However, the Entropy index exhibited a declining trend, suggesting that the classification accuracy declined with increasing numbers of trajectories. Notably, when the trajectory count increased from two to three, substantial declines in the absolute values of AIC and BIC were observed, suggesting significant improvements in the goodness of fit. By contrast, when expanding the trajectory count from three to four and subsequently from four to five, the magnitude of these declines diminished considerably, suggesting limited enhancements in the goodness of fit. Also, existing studies have indicated that the LMR-LRT tends to exhibit overfitting in large samples and should only be used as a subordinate reference when determining the number of latent trajectories (Nylund et al., 2007). Given the sample size of 16,659 in this study, relying solely on LMR-LRT for group number selection would clearly introduce significant bias. Furthermore, previous research recommends that the Entropy index should not fall below 0.80 (C. S. Lee et al., 2020). The results demonstrate that while the Entropy index shows only a slight decrease when increasing from 2 to 3 groups and remains above the 0.80 threshold, it experiences a substantial decline when expanding from 3 to 4 groups, dropping below 0.80. This suggests insufficient classification accuracy for the four-group solution (and also for the five-group solution). Considering these indicators together with the substantive interpretability of the trajectory groups, we ultimately selected the three-group solution as the optimal model. More details on the reliability of the three−trajectory model can be observed in Table A1.

**Figure 1:**
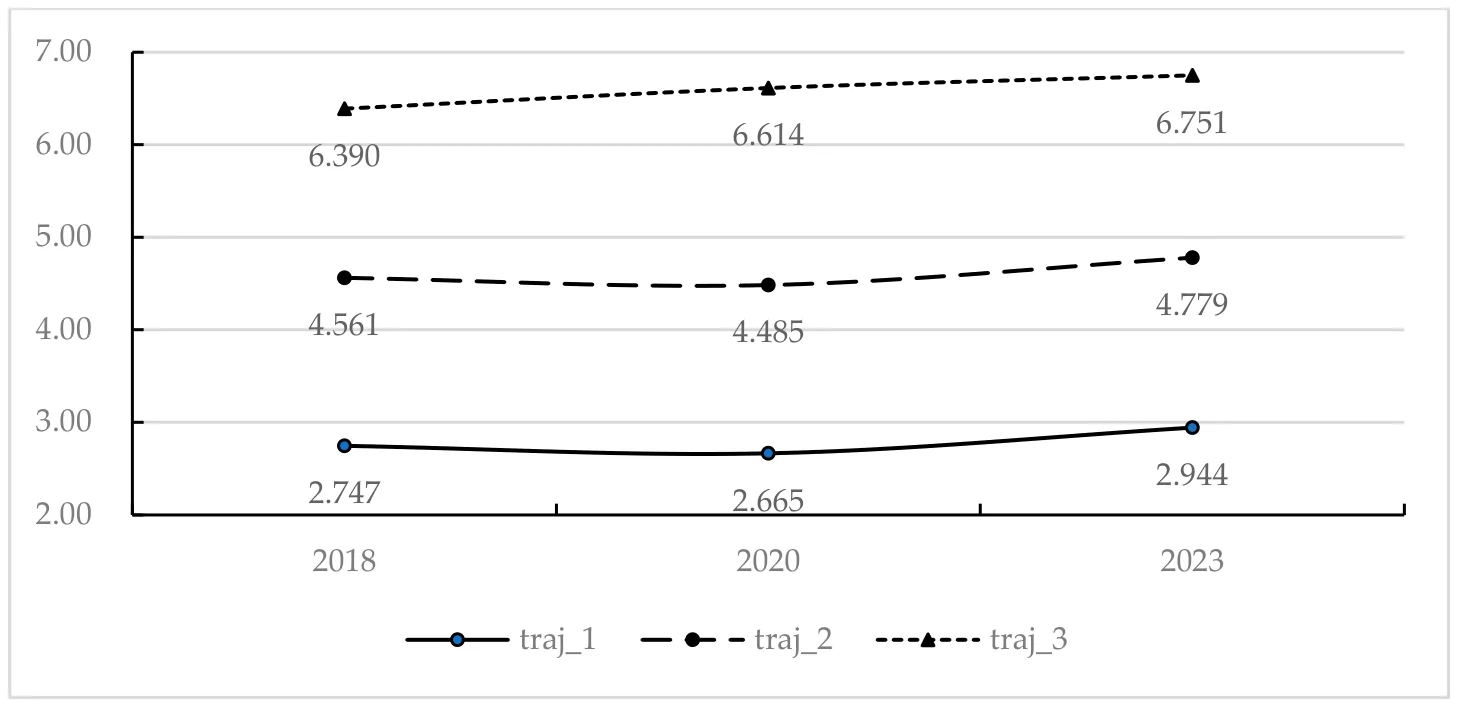
Changes in three housing quality trajectories.

Figure 1 presents the specific characteristics of each group-based trajectory. Trajectory 1 accounted for 15.3% of the total sample (n = 851), with a posterior probability of 16.3% and an intra-trajectory average posterior probability of 94.5% (>70% indicates acceptable classification accuracy). This group exhibited the lowest level of housing quality, demonstrating a V-shaped fluctuating trajectory characterized by an initial decline followed by an increase (linear coefficient = −0.332, *p* < 0.001; quadratic coefficient = 0.003, *p* < 0.001), so we labeled it as the “low-fluctuating growth” trajectory. Trajectory 2 constituted 30.3% of the total sample (n = 1685), with a posterior probability of 30.0% and an intra-trajectory average posterior probability of 88.5%. This group exhibited a moderate level of housing quality, demonstrating a V-shaped fluctuating trajectory, displaying a similar V-shaped variation pattern (linear coefficient = −0.225, *p* = 0.001; quadratic coefficient = 0.002, *p* < 0.001), so we labeled it as the “moderate-fluctuating growth” trajectory. Trajectory 3 accounted for 54.3% of the total sample (n = 3017), with a posterior probability of 53.7% and an intra-trajectory average posterior probability of 95.8%. This group exhibited the highest level of housing quality with no significant linear or quadratic change with period (linear coefficient = 0.055, *p* = 0.236; quadratic coefficient = −0.000, *p* = 0.297), so we labeled it as the “consistently high-level” trajectory.

### 4.3. Associations Between Housing Quality Trajectories and Elderly Depression

Table 3 presents the association between housing quality trajectories and depression in Chinese older adults. Model 1 demonstrates that, compared to trajectory 1, trajectory 2 exhibited no significant difference in depression levels, while trajectory 3 exhibited significantly lower depression levels (β = −0.484, *p* < 0.001). That is, during the period of 2018–2023, older adults who consistently maintained high-level housing quality exhibited significantly lower depression levels compared to those with low-level housing quality characterized by fluctuating growth; older adults with moderate-level housing quality characterized by fluctuating growth showed no significant difference in depression levels relative to those with low-level housing quality characterized by fluctuating growth. Thus, H1 was supported.

Models 2 and 3 reveal negative associations between consistently high housing quality levels and reduced elderly depression in rural areas. Although the estimated coefficient for trajectory 3 was significant (*p* < 0.01) in rural areas and non-significant in urban areas (*p* > 0.05), the coefficient difference test suggests no significant difference between them. Thus, H2 was not supported.

### 4.4. Mediation Analysis

Table 4 reports the potential mediation effects of physical health, social support networks, and social adaptation. The results indicate that in the total sample, both social support networks and social adaptation significantly mediated the positive association between consistently high-level housing quality trajectory and reduced elderly depression; their mediation effects (β = −0.042, *p* < 0.001; β = −0.041, *p* < 0.001; respectively) accounted for 8.7% and 8.5% of the total effect (β = −0.484, *p* < 0.001), respectively. However, physical health showed a significant shading effect (β = 0.029, *p* < 0.05); better physical health shaded the positive association between consistently high-level housing quality trajectory and reduced elderly depression. Accordingly, H3b and H3c were supported, while H3a was not supported.

The aforementioned mechanisms exhibited obvious urban–rural differences. In urban areas, physical health showed a significant shading effect (β = 0.172, *p* < 0.001), and better physical health shaded the positive association between consistently high-level housing quality trajectory and reduced elderly depression; however, social support networks and social adaptation did not show a significant mediating effect. By contrast, in rural areas, physical health, social support networks, and social adaptation significantly mediated the positive association between consistently high-level housing quality trajectory and reduced elderly depression; their mediation effects (β = −0.061, *p* < 0.01; β = −0.067, *p* < 0.01; β = −0.066, *p* < 0.01; respectively) accounted for 12.8%, 14.0%, and 13.8% of the total effect (β = −0.478, *p* < 0.01), respectively.

### 4.5. Robustness Tests

Individuals with more mental health risks are more likely to migrate to or remain in poor residential conditions (Sanders et al., 2025a), indicating a selection bias. Thus, the propensity score matching (PSM) method was used to mitigate the selection bias. Panel A in Table A2 suggests that only consistent high-level trajectory (trajectory 3) was significantly associated with reduced elderly depression (ATE = −0.502, *p* < 0.01), which is consistent with the OLS findings in Table 3.

To enhance the clinical relevance of the analysis regarding elderly depression, we dichotomized the continuous depression variable based on prior research (Jiang & Wang, 2020), creating a binary outcome indicating the presence of depressive symptoms (scores of 0~11 coded as 0 meant no; scores of 12~18 coded as 1 meant yes). Based on the binary Logit model, Panel B in Table A2 reports a result that is highly similar to that in Table 3, suggesting its robustness.

To address the confounding of time-invariant individual heterogeneity, we conducted a supplemented test using the fixed effect (FE) model. Panel C in Table A2 demonstrates that after controlling for time-invariant individual characteristics, changes in housing quality were negatively related to variations in elderly depression, with this association being statistically significant in both urban and rural contexts^4^. These findings confirm the robustness of the aforementioned results.

Finally, to test the sensitivity of the KHB mediation results in Table 4, we further used the causal mediation analysis to conduct a robustness test. Table A3 reports a result that was highly similar to that in Table 4, suggesting its robustness.

## 5. Discussion

Based on the nationally representative longitudinal data, this study identified three trajectories of housing quality among Chinese older adults: “consistently high-level”, “moderate-fluctuating growth” and “low-fluctuating growth”. Compared to the “low-fluctuating growth” trajectory, older adults in the “consistently high-level” trajectory exhibited significantly lower depression levels (small to medium effect size), whereas the “moderate-fluctuating growth” trajectory showed no significant difference. This association did not differ significantly between urban and rural areas. However, in rural areas, better physical health, stronger social support networks, and higher levels of social adaptation served as important mediators that linked elevated housing quality with reduced depression. By contrast, in urban areas, social support networks and social adaptation did not demonstrate significant mediating effects, and better physical health may mask the association between housing quality and elderly depression.

Firstly, only the elderly with long-term access to better residential conditions demonstrate significantly lower depression levels, whereas moderate or poor housing quality, as well as phased declines in housing quality over time, may lead to increased depression risks—a finding consistent with prior research (Liang, 2020; Szabo et al., 2018). This indicates that the persistence and stability of housing quality are critical for elderly mental health, representing a potential intervention direction for promoting psychological well-being in the elderly. While phased improvements or enhancements in housing quality may not effectively counteract the cumulative depressive risks associated with long-term disadvantageous living conditions, it is noteworthy that two trajectory groups both experienced phased declines in housing quality in 2020, which may be partially associated with the outbreak of the COVID-19 pandemic at the beginning of that year. Affected by the pandemic, China’s economic growth rate slowed significantly while unemployment rates increased (Cai et al., 2021), resulting in specific population groups being unable to obtain sufficient economic returns to maintain their previous housing conditions.

Secondly, an important reason why better housing conditions are associated with lower levels of depression is that they are typically linked to enhanced social adaptation and more extensive social support networks, which resonates with the prior literature on social withdrawal. Wells and Harris (2007) observed that adverse housing conditions were related to reduced social interactions and weakened social adaptation in female groups, triggering social withdrawal phenomena that exacerbated psychological distress. By contrast, improved housing conditions function as facilitators for social participation and interpersonal interactions (Song et al., 2025; Szabo et al., 2018). This study further identified similar mechanisms among the elderly population, extending and deepening the theoretical framework of social withdrawal. Notably, the mediation effects of social support networks and social adaptation were only significant among rural seniors, indirectly corroborating Liang’s (2020) findings. Liang (2020) highlighted that rural China features closer interpersonal relationships where housing serves as a crucial venue for social interactions while simultaneously carrying psychosocial attributes of “flaunt”. Thus, housing characteristics may uniquely relate to elderly depression through these context-specific mechanisms. By contrast, urban elderly populations typically maintain more dispersed social networks, where the association between housing conditions and social support networks might be weaker.

It is noteworthy that the potential mechanism of physical health exhibits distinct pathways in urban and rural areas. Among rural elderly populations, physical health mediates the association between improved housing quality and reduced depression, while it masks the association among urban elderly populations. A possible explanation lies in our operationalization of physical health using chronic disease prevalence and physical pain indicators. Given the general improvement of housing quality in both urban and rural China in recent years, disparities in housing quality primarily manifest in the adequacy of indoor living facilities rather than extreme poor conditions characterized by pollution or uneven floors. Consequently, the impact of housing quality on physical health remains weak, becoming evident only among certain rural populations with relatively poor housing conditions. In addition, the intrinsic driving mechanisms behind housing quality improvement may contribute to this phenomenon. Given that the promotion of housing quality is strongly policy-driven in rural China (Liang, 2020; Xiao & Selvaratnam, 2024), such promotion may represent external economic resource supplements that improve physical health without imposing additional burdens on elderly populations, thereby reducing their depression levels. By contrast, the promotion of housing quality demonstrates stronger market-driven characteristics in urban China (Liang, 2020; Xiao & Selvaratnam, 2024). Better housing conditions among urban elderly populations often results from prolonged work (or even overwork) throughout previous decades. This accumulation of chronic health risks means that better housing conditions do not necessarily translate into better physical health for urban seniors, thus masking the expected association between improved housing quality and declined depression.

These findings carry some policy implications. First, given that the persistence and stability of housing quality may be a critical protective factor for elderly mental health, policymakers should prioritize ensuring long-term, stable enhancement of housing quality for older adults, establishing robust housing quality monitoring and early warning systems to prevent seniors from experiencing housing quality deterioration caused by economic fluctuations, natural disasters, or other factors. Second, considering the critical mediation role of social adaptation and social support networks in rural elderly populations, housing quality improvement policies in rural areas should emphasize the socializing function of housing—such as expanding public activity spaces and optimizing aging-friendly designs to facilitate social interactions among rural seniors. Finally, particular attention should be paid to the differential role of physical health between urban and rural elderly populations. It is essential to actively promote rural dilapidated housing renovation and housing quality monitoring projects to prevent extreme housing conditions from endangering the physical health of rural seniors; also, equal emphasis should be placed on urban elderly residents who maintain good or moderate housing quality but suffer from poor physical health status, providing appropriate health safety net services for them.

There were still some notable limitations to this study. First, the estimates based on the OLS model could not establish causal relationships between housing quality trajectories and depression among older adults, although a series of robustness checks have yielded consistent results. Second, using three-wave longitudinal data, only three distinct longitudinal change patterns of housing quality were identified, which failed to capture more diverse and nuanced temporal trajectories; restricting the sample to participants who completed all three survey waves might also introduce selection bias. Third, the measurement of housing quality was limited to indoor environments and household facilities, without comprehensively considering other housing conditions such as neighborhood environments and perceptions of housing quality. Finally, although the GBTM demonstrated an acceptable model fit, potential misclassification errors might still exist in our analyses.

## 6. Conclusions

The Chinese elderly population demonstrates three distinct longitudinal trajectories of housing quality. Compared to the “low-fluctuating growth” trajectory, older adults in the “consistently high-level” trajectory exhibited significantly lower depression levels, whereas the “moderate-fluctuating growth” trajectory showed no significant difference. In rural areas, physical health, social support networks, and social adaptation served as important mediators that linked elevated housing quality with reduced depression, while in urban areas, social support networks and social adaptation did not demonstrate significant mediating effects, and physical health may mask the association between housing quality and elderly depression. These findings transcend the limitations of most previous studies that focused solely on static housing conditions and reveal the differential mechanisms through which housing quality trajectories relate to elderly depression, as well as the urban–rural disparities therein. This suggests that we should avoid staged declines in housing quality among the elderly and pay attention to urban–rural differences in the mechanisms linking housing quality trajectories with elderly depression.

## Figures and Tables

**Table 1:** Descriptive statistics of the samples, N = 16,659.

Variables	Urban (n = 9229)		Rural (n = 7430)		*p*-Value of *t* Test or chi2 Test	Effect Size (Cohen’s d or Cohen’s w)
	n (%)	Mean ± SD	n (%)	Mean ± SD		
Sex					<0.001	0.059
Female	4848 (52.5)		3464 (46.6)			
Male	4381 (47.5)		3966 (53.4)			
Age		69.50 ± 5.15		70.02 ± 5.23	<0.001	0.099
Schooling year		7.02 ± 3.94		4.53 ± 3.78	<0.001	0.614
Marital status					0.001	0.025
Married	7432 (80.5)		5834 (78.5)			
Unmarried	1797 (19.5)		1596 (21.5)			
Living alone					0.006	0.021
No	8408 (91.1)		6676 (89.9)			
Yes	821 (8.9)		754 (10.1)			
Physical health		1.46 ± 0.82		1.39 ± 0.82	<0.001	0.078
Social support networks		13.92 ± 5.01		14.29 ± 5.41	<0.001	0.070
Social adaptation		16.66 ± 4.10		15.91 ± 3.86	<0.001	0.186
Housing quality		6.14 ± 1.34		4.49 ± 1.65	<0.001	0.969
Depression score		6.30 ± 3.08		7.14 ± 2.91	<0.001	0.256

**Table 2:** The goodness of fit of GBTMs.

Number of Trajectories	AIC	BIC	log-Likelihood	Entropy	*p*-Value of LMR-LRT
2	−28,544.83	−28,571.32	−28,536.83	0.874	
3	−27,749.55	−27,789.28	−27,737.55	0.839	<0.001
4	−27,534.92	−27,587.90	−27,518.92	0.744	<0.001
5	−27,435.37	−27,501.59	−27,415.37	0.766	<0.001

**Table 3:** Associations between housing quality trajectories and elderly depression based on OLS model.

	Model 1: Total Sample		Model 2: Urban		Model 3: Rural	
	β	Robust SE	β	Robust SE	β	Robust SE
Housing quality (reference: trajectory 1)						
trajectory 2	−0.061	0.122	−0.005	0.244	−0.061	0.141
trajectory 3	−0.484 ***	0.128	−0.429	0.225	−0.478 **	0.168
Sex (reference: female)	−0.292 ***	0.085	−0.373 **	0.115	−0.199	0.127
Age	0.063 ***	0.008	0.057 ***	0.012	0.071 ***	0.012
Residence (reference: rural)	−0.464 ***	0.097				
Marital status (reference: married)	0.395 ***	0.110	0.354 *	0.155	0.433 **	0.155
Schooling year	−0.053 ***	0.011	−0.067 ***	0.015	−0.034 *	0.017
Living alone (reference: no)	−0.188	0.157	−0.209	0.223	−0.163	0.220
Intercept	3.360 ***	0.582	3.413 ***	0.825	2.666 **	0.819
R-squared	0.048		0.031		0.031	
n	5553		3137		2416	

Note: * *p* < 0.05, ** *p* < 0.01, and *** *p* < 0.001. SE represents standard errors. The coefficient difference test using the Stata command “bdiff” suggested no significant difference in the associations of interest between urban and rural older adults. The effect size of the association between housing quality trajectories and elderly depression was small to medium in the total sample (Cohen’s f = 0.130), while it was small in both urban and rural samples (Cohen’s f = 0.070 and 0.071, respectively).

**Table 4:** Mediation analysis based on the KHB method.

		Model 1: Total Sample		Model 2: Urban		Model 3: Rural	
		β	Robust SE	β	Robust SE	β	Robust SE
Trajectory 2	Total effect	n.s.		n.s.		n.s.	
	Direct effect	n.s.		n.s.		n.s.	
	Mediation effect	n.s.		n.s.		n.s.	
Trajectory 3	Total effect	−0.484 ***	0.126	−0.429	0.225	−0.478 **	0.165
	Direct effect	−0.429 ***	0.128	−0.586 *	0.231	−0.284	0.167
	Mediation effect						
	Physical health	0.029 *	0.012	0.172 ***	0.044	−0.061 **	0.020
	Social support networks	−0.042 ***	0.012	−0.009	0.010	−0.067 **	0.021
	Social adaptation	−0.041 ***	0.013	−0.007	0.020	−0.066 **	0.024

Note: * *p* < 0.05, ** *p* < 0.01, and *** *p* < 0.001. SE represents standard errors. All other covariates were controlled for in all three models.

## Data Availability

The data presented in this study are available upon request from the corresponding author due to privacy restrictions.

## Notes

1 We compared the proportions or mean values of samples before and after missing data imputation across variables, including sex, urban–rural residence, schooling year, and self-rated health, and found no substantive differences. This suggests that the missingness can be considered random, and thus, the sample loss does not significantly compromise the fundamental structure of the dataset.

2 Prior research documents that when a concept is defined in a formative rather than a reflective way, its reliability coefficient is usually low (Ke et al., 2019). Evidently, the nine items used to measure housing quality are formative indicators, so a reliability coefficient of 0.655 is acceptable.

3 For the GBTM, the Stata command “traj” was used; for the KHB analysis, the Stata command “khb” was used; for the PSM analysis, the Stata command “teffects” was used; and for the causal mediation analysis, the Stata command “medeff” was used.

4 It should be noted that the association between housing quality change and changes in elderly depression in the urban sample was statistically significant at the 0.01 level. A reasonable explanation is that the non-significant results presented in Table 3 are mainly caused by higher heterogeneity within the urban elderly group, and the FE model precisely controlled this heterogeneity, thus obtaining such a significant estimation.

## Abbreviations

The following abbreviations are used in this manuscript:

AIC	Akaike Information Criterion
ATE	Average treatment effect
BIC	Bayesian Information Criterion
CLASS	China Longitudinal Aging Social Survey
CES-D	Center for Epidemiologic Studies Depression
FE	Fixed effect
GBTM	Group-based trajectory model
KHB	Kristian–Holm–Breen
LMR-LRT	Lo–Mendell–Rubin likelihood ratio test
OLS	Ordinary least squares
PSM	Propensity score matching
SD	Standard deviation
SE	Standard error

## Appendix A

**Table A1:** Reliability of the three-trajectory model.

	Trajectory	Criterion	Estimate	Is It Acceptable?
Entropy		>0.80	0.839	yes
Average posterior probability per trajectory	trajectory 1	>0.70	0.945	yes
	trajectory 2	>0.70	0.885	yes
	trajectory 3	>0.70	0.958	yes
Odds of correct classification	trajectory 1	>5.00	88.25	yes
	trajectory 2	>5.00	17.70	yes
	trajectory 3	>5.00	19.20	yes
Difference between trajectory size and posterior probability	trajectory 1	<0.05	0.010	yes
	trajectory 2	<0.05	0.004	yes
	trajectory 3	<0.05	0.006	yes

**Table A2:** Results based on PSM methods, binary depressive outcome, and FE models.

**Panel A: PSM**	**Model 1: Total Sample**		**Model 2: Urban**		**Model 3: Rural**	
	**ATE**	**Robust SE**	**ATE**	**Robust SE**	**ATE**	**Robust SE**
Housing quality (reference: trajectory 1)						
trajectory 2	−0.063	0.172	0.007	0.291	−0.046	0.143
trajectory 3	−0.502 **	0.166	−0.422	0.270	−0.503 **	0.171
Control variables	Yes		Yes		Yes	
N	5553		3137		2416	
**Panel B: Binary Outcome**	**Model 4: Total Sample**		**Model 5: Urban**		**Model 6: Rural**	
	**β**	**Robust SE**	**β**	**Robust SE**	**β**	**Robust SE**
Housing quality (reference: trajectory 1)						
trajectory 2	−0.053	0.175	0.319	0.455	−0.087	0.197
trajectory 3	−0.376 *	0.191	−0.008	0.441	−0.430 *	0.216
Control variables	Yes		Yes		Yes	
Pseudo R-squared	0.014		0.018		0.013	
Observations	5553		3137		2416	
**Panel C: FE**	**Model 7: Total Sample**		**Model 8: Urban**		**Model 9: Rural**	
	**β**	**Robust SE**	**β**	**Robust SE**	**β**	**Robust SE**
Housing quality	−0.136 ***	0.033	−0.171 **	0.053	−0.136 **	0.047
Control variables	Yes		Yes		Yes	
R-squared	0.057		0.027		0.020	
Observations	16,659		9229		7430	

Note: * *p* < 0.05, ** *p* < 0.01, and *** *p* < 0.001. SE represents standard errors. ATE represents average treatment effect. Control variables included sex, age, residence, marital status, schooling year, and living alone. The balance test suggests that the standard bias of all covariates between trajectory 1 and trajectories 2 and 3 was less than 0.10 after matching, suggesting a good balance in the PSM method (Jiang & Wang, 2020). The Rosenbaum bounds sensitivity analysis exhibits that the *p*-value was still smaller than 0.05 when gamma equaled two, suggesting non-sensitive PSM results.

**Table A3:** Results of causal mediation analysis.

		Model 1		Model 2		Model 3	
		β	SE	β	SE	β	SE
Trajectory 2	Total effect	n.s.		n.s.		n.s.	
	Direct effect	n.s.		n.s.		n.s.	
	Mediation effect	n.s.		n.s.		n.s.	
Trajectory 3	Total effect	−0.486 ***	0.116	−0.486 ***	0.123	−0.486 ***	0.120
	Direct effect	−0.519 ***	0.128	−0.444 ***	0.129	−0.440 ***	0.129
	Mediation effect						
	Physical health	0.033 *	0.013				
	Social support networks			−0.042 ***	0.011		
	Social adaptation					−0.046 ***	0.011

Note: * *p* < 0.05, ** *p* < 0.01, and *** *p* < 0.001. SE represents standard errors.

## References

[B1-behavsci-16-01920] Baek S.-U., Yoon J.-H. (2025). Association of housing precariousness with depressive symptoms and suicidal ideation: Findings from a nationwide survey of young adults in South Korea. Health & Place.

[B2-behavsci-16-01920] Cai F., Zhang D., Liu Y. (2021). The impact of COVID-19 on the Chinese labor market: A comprehensive analysis based on the individual tracking survey. Economic Research Journal.

[B3-behavsci-16-01920] Charlson F. J., Baxter A. J., Cheng H. G., Shidhaye R., Whiteford H. A. (2016). The burden of mental, neurological, and substance use disorders in China and India: A systematic analysis of community representative epidemiological studies. Lancet.

[B4-behavsci-16-01920] Chen K., Wang W., Qiu J., Guo W., Du J., Gao B., Hu Z., Qiao H. (2024). Housing conditions, cooking fuels, and health-related quality of life among rural middle-aged and elderly in northwest China: A ten-year balanced panel study. Preventive Medicine Reports.

[B5-behavsci-16-01920] Cheng H., Wu Y., Huang S., Zhang S., Zhang J., Wu B., Zhang J. (2025). Focus on housing issues: Associations between housing quality and cognitive impairment among older adults in China and India. Journal of Alzheimer’s Disease.

[B6-behavsci-16-01920] Garg R., McQueen A., Wolff J. M., Skinner K. E., Kegler M. C., Kreuter M. W. (2022). Low housing quality, unmet social needs, stress and depression among low-income smokers. Preventive Medicine Reports.

[B7-behavsci-16-01920] He H., Yan C. (2022). Segregation or integration: A study of the impact of Internet use on community participation of Chinese elderly. Population Journal.

[B8-behavsci-16-01920] Jiang J., Deng X. (2026). Influence of social relationship disruption on the depression of Chinese older adults: A study based on the China Longitudinal Aging Social Survey, 2018–2023. Journal of Gerontological Social Work.

[B9-behavsci-16-01920] Jiang J., Luo L. (2024). Does Internet use improve the physical and mental health of older adults in China? A study based on the digital divide theory. Geriatric Nursing.

[B10-behavsci-16-01920] Jiang J., Wang P. (2020). Does early peer relationship last long? The enduring influence of childhood peer relationship on depression in middle and later life. Journal of Affective Disorders.

[B11-behavsci-16-01920] Kang N., You J., Huang J., Ren Y., Lin M., Xu S. (2019). Understanding the pathways from depression to suicidal risk from the perspective of the interpersonal-psychological theory of suicide. Suicide and Life-Threatening Behavior.

[B12-behavsci-16-01920] Karlson K. B., Holm A., Breen R. (2012). Comparing regression coefficients between same-sample nested models using Logit and Probit: A new method. Sociological Methodology.

[B13-behavsci-16-01920] Ke Y., Jiang J., Chen Y. (2019). Social capital and the health of left-behind older adults in rural China: A cross-sectional study. BMJ Open.

[B14-behavsci-16-01920] Lee C. S., Faulkner K. M., Thompson J. H. (2020). Identifying subgroups: Part 2: Trajectories of change over time. European Journal of Cardiovascular Nursing.

[B15-behavsci-16-01920] Lee J. H. (2024). Housing quality determinants of depression and suicide ideation by age and gender. Housing Studies.

[B16-behavsci-16-01920] Li T., Zhang Y. (2014). Growth curve trajectories of elderly people’s health indicators in China: Cohort variations and rural-urban disparities. Population Research.

[B17-behavsci-16-01920] Liang Y. (2020). Heterogeneity in the trajectories of depressive symptoms among elderly adults in rural China: The role of housing characteristics. Health & Place.

[B18-behavsci-16-01920] Liang Y., Hou B., Li S. (2017). The influence of living pressure and living conditions on the mental health of migrant workers. Urban Problems.

[B19-behavsci-16-01920] McElroy B., Walsh E. (2023). A happy home? Socio-economic inequalities in depressive symptoms and the role of housing quality in nine European countries. BMC Public Health.

[B20-behavsci-16-01920] Morganti A., Brambilla A., Aguglia A., Amerio A., Miletto N., Parodi N., Porcelli C., Odone A., Costanza A., Signorelli C., Serafini G., Amore M., Capolongo S. (2022). Effect of housing quality on the mental health of university students during the COVID-19 lockdown. International Journal of Environmental Research and Public Health.

[B21-behavsci-16-01920] Nagin D. S. (2005). Group-based modeling of development.

[B22-behavsci-16-01920] Nagin D. S., Odgers C. L. (2010). Group-based trajectory modeling in clinical research. Annual Review of Clinical Psychology.

[B23-behavsci-16-01920] Nylund K. L., Asparouhov T., Muthén B. O. (2007). Deciding on the number of classes in latent class analysis and growth mixture modeling: A Monte Carlo simulation study. Structural Equation Modeling.

[B24-behavsci-16-01920] Osuna M., Jaramillo A., Ailshire J. (2026). The relationship between housing, neighborhood and community infrastructure and older adult depression in Colombia. Wellbeing, Space and Society.

[B25-behavsci-16-01920] Pevalin D. J., Reeves A., Baker E., Bentley R. (2017). The impact of persistent poor housing conditions on mental health: A longitudinal population-based study. Preventive Medicine.

[B26-behavsci-16-01920] Pineda J. C. D., Kokubun K., Ikaga T., Yamakawa Y. (2021). Housing quality and behavior affect brain health and anxiety in healthy Japanese adults. Scientific Reports.

[B27-behavsci-16-01920] Roberts M. K., Bhat A. C., Fenelon A. (2025). The long-term effects of housing insecurity in young adulthood on subsequent material hardship, physiological and mental health. Social Science & Medicine.

[B28-behavsci-16-01920] Sanders F., Baltramonaityte V., Lussier A. A., Smith A. D., Dunn E. C., Walton E. (2025a). Home and epigenome: Exploring the role of DNA methylation in the relationship between poor housing quality and depressive symptoms. BMJ Public Health.

[B29-behavsci-16-01920] Sanders F., Waldren L. H., Baltramonaityte V., Lussier A. A., Walton E. (2025b). A network analysis of housing quality indicators and depression in women. Scientific Reports.

[B30-behavsci-16-01920] Schilz L., Kemna S., Karnouk C., Böge K., Lindheimer N., Walther L., Mohamad S., Suboh A., Hasan A., Höhne E., Banaschewski T., Plener P., Strupf M., Hahn E., Bajbouj M. (2023). A house is not a home: A network model perspective on the dynamics between subjective quality of living conditions, social support, and mental health of refugees and asylum seekers. Social Psychiatry and Psychiatric Epidemiology.

[B31-behavsci-16-01920] Snedker K. A., Herting J. R. (2016). Adolescent mental health: Neighborhood stress and emotional distress. Youth & Society.

[B32-behavsci-16-01920] Song J., Zuo W., Zhuang X., Wu R. (2025). How do housing quality and environmental pollution affect older adults’ self-rated health? The mediating effect of depression and social capital. Buildings.

[B33-behavsci-16-01920] Szabo A., Allen J., Alpass F., Stephens C. (2018). Longitudinal trajectories of quality of life and depression by housing tenure status. Journals of Gerontology: Social Sciences.

[B34-behavsci-16-01920] Taylor N. C., Woodhall-Melnik J., Lamont A., Dunn J. R., Nombro E., Dutton D. J. (2026). The impact of place on health: Neighbourhood & residential satisfaction as a contributor to physical and mental health in applicants for publicly subsidized housing. Health & Place.

[B35-behavsci-16-01920] Wang X., Sun Q., Du X., Rong J. (2024). Association between activity of daily living and depressive symptoms among older adults in China: Evidence from the CHARLS. Journal of Public Health.

[B36-behavsci-16-01920] Wells N. M., Harris J. D. (2007). Housing quality, psychological distress, and the mediating role of social withdrawal: A longitudinal study of low-income women. Journal of Environmental Psychology.

[B37-behavsci-16-01920] Wen Y., Lian L. (2025). Digital empowerment differentiation: The moderating effect of education level on the relationship between older adults’ Internet use and social adaptation. Population and Society.

[B38-behavsci-16-01920] Wong C. S. M., Chan W. C., Chu N. W. T., Law W. Y., Tang H. W. Y., Wong T. Y., Chen E. Y. H., Lam L. C. W. (2024). Individual and interactive effects of housing and neighborhood quality on mental health in Hong Kong: A retrospective cohort study. Journal of Urban Health.

[B39-behavsci-16-01920] Wong M. Y. C., Ou K.-l., Chung P. K., Chui K. Y. K., Zhang C.-Q. (2023). The relationship between physical activity, physical health, and mental health among older Chinese adults: A scoping review. Frontiers in Public Health.

[B40-behavsci-16-01920] Xiao Y., Selvaratnam D. P. (2024). Impact of rural housing reform on economic growth and quality of life in China: An analysis from 1998 to 2022. International Journal of Social Science and Human Research.

[B41-behavsci-16-01920] Xu J., Cao X. (2025). The dynamic relationship between cognitive decline and depression in older adults and the moderating role of social participation. Chinese Journal of Population Science.

[B42-behavsci-16-01920] Yuan H. (2025). The mediating effect of subjective housing quality on the relationship between housing conditions and mental health: Evidence from China’s mega-cities. Behavioral Sciences.

[B43-behavsci-16-01920] Yuan M. (2022). The impact of urban residential aging-adaptation renovations on the health of elderly living at home: An analysis based on hierarchical linear models. Urban Problems.

[B44-behavsci-16-01920] Zhang P., Zhong J., Hu Y. (2023). The health-enhancing effects of the implementation of rural dilapidated house renovation policy. Chinese Rural Economy.

[B45-behavsci-16-01920] Zhou Z., Ma Y., Du W., Zhou K., Qi S. (2022). Housing conditions and adolescents’ socioemotional well-being: An empirical examination from China. Applied Research in Quality of Life.

